# Diphtheria

**Published:** 1882-12

**Authors:** 


### DIPHTHERIA.

From the Board of Health, we learn that diphtheria has, within the past two months, become quite prevalent in this city.

One of the most terrible of all diseases, it has succeeded so far in eluding the penetrating grasp of science, and we really know no more about it to-day, that is to say, about its causation and prevention, than we did two decades ago.

It was with the greatest pleasure and pride that we published, last spring, the report of Professor Wood and Dr. Formad, detailing their experience with an epidemic of diphtheria at Ludington, Mich.

They concluded that the disease was caused by a specific micrococcus, and the National Board of Health endorsed their conclusions.

But now, this very same Dr. Formad has demolished his own conclusions. Within six short months since his results were published, he has declared them untrue.

Still, this apparent contradiction of facts will, we doubt not, bear good fruit, for, from what we can learn, it seems certain that these bacilli and micrococci (about which we hear so much), have an important bearing on the production of disease, even though they do not produce the disease per se.

In the meantime, until we learn something more about diphtheria, we must carefully *isolate*, *disinfect* and treat to the best of our limited ability.—*Med. and Surg. Rep.*

